# Bilineal inheritance of pathogenic *PKD1* and *PKD2* variants in a Czech family with autosomal dominant polycystic kidney disease – a case report

**DOI:** 10.1186/s12882-018-0978-2

**Published:** 2018-07-04

**Authors:** Veronika Elisakova, Miroslav Merta, Jana Reiterova, Alica Baxova, Jaroslav Kotlas, Katerina Hirschfeldova, Lena Obeidova, Vladimir Tesar, Jitka Stekrova

**Affiliations:** 1 Institute of Biology and Medical Genetics, First Faculty of Medicine Charles University and General University Hospital in Prague, Albertov 4, 128 00 Prague, Czech Republic; 2 Department of Nephrology, First Faculty of Medicine Charles University and General University Hospital in Prague, U Nemocnice 2, 128 00 Prague, Czech Republic

**Keywords:** ADPKD, *PKD1/2* gene, Bilineal inheritance, Causative mutation, Modifier gene

## Abstract

**Background:**

Autosomal dominant polycystic kidney disease (ADPKD) is the most common hereditary renal disorder, leading to end stage renal failure and kidney transplantation in its most serious form. The severity of the disease’s manifestation depends on the genetic determination of ADPKD. The huge variability of different phenotypes (even within a single family) is not only modulated by the two main ADPKD genes (*PKD1* and *PKD2*) but also by modifier genes and the whole genetic background.

**Case presentation:**

This is a report of an ADPKD family with co-inheritance of *PKD1* and *PKD2* pathogenic variants. The proband, with an extremely serious manifestation of ADPKD (the man was diagnosed in early childhood, and with end stage renal disease aged 23), underwent genetic analysis of *PKD1* and *PKD2*, which revealed the presence of pathogenic mutations in both of these genes. The missense *PKD2* mutation p.Arg420Gly came from the proband’s father, with a mild ADPKD phenotype. The same mutation of the *PKD2* gene and similar mild disease presentation were found in the proband’s aunt (father’s sister) and her son. The nonsense mutation p.Gln2196* within the *PKD1* gene was probably inherited from the proband’s mother, who died at the age of 45. It was only discovered post mortem, that the real cause of her death was kidney failure as a consequence of untreated ADPKD. Unfortunately, neither the DNA of the proband’s mother nor the DNA of any other family members from this side of the pedigree were available for further examination. The proband underwent successful cadaveric kidney transplantation at the age of 24, and this replacement therapy lasted for the next 15 years.

**Conclusions:**

Here, we present a first case of bilineal ADPKD inheritance in the Czech Republic. This report highlights the significant role of modifier genes in genetic determination of ADPKD, especially in connection with seriously deteriorated disease phenotypes. In our case, the modifying role is probably mediated by the *PKD2* gene.

## Background

Autosomal dominant polycystic kidney disease (ADPKD; OMIM *601313 for *PKD1*, and *173910 for *PKD2*) is the most common hereditary kidney disease, predominantly characterized by the presence of cysts in both kidneys leading to end-stage renal disease (ESRD), usually in adulthood [1]. Its incidence is 1 per 400–1000 persons in the general population. The systemic and multi-organ nature of ADPKD manifests by the occurrence of cysts in extrarenal organs (liver, pancreas, spleen), as well as by other manifestations such as cardiovascular abnormalities and/or brain vessel aneurysms [2, 3]. Cystogenesis is associated with the development of arterial hypertension, which precedes the deterioration of the glomerular filtration rate in a notable proportion of ADPKD patients. Alterations in epithelial cell growth, fluid secretion, and extracellular matrix composition are the main pathophysiological abnormalities observed during the onset and growth of the cysts. These processes are caused by the deregulation of cAMP, as well as EGFR- and mTOR-mediated pathways. Two genes (*PKD1* and *PKD2*) have been uncovered as being causative in ADPKD [4, 5]. *PKD1* and *PKD2* code for two gene products: polycystin 1 (PC-1) and polycystin 2 (PC-2), respectively. The germline mutations in *PKD1* are present in approximately 85% of the ADPKD patients (ADPKD type 1); mutations in *PKD2* in the remaining 15% of ADPKD patients (ADPKD type 2). However, the portion of *PKD2* mutations can rise to 36% in community-based studies [6]. Clinical manifestations due to defective PC-1 in the *PKD1* genotype and PC-2 in the *PKD2* genotype display a phenotypic similarity, which is largely explained by the interaction between both polycystins. PC-1 and PC-2, together with fibrocystin/polyductin, which is responsible for autosomal recessive polycystic kidney disease (ARPKD), form a complex on the surface of the bottom part of the primary cillium. Defects of PC-1 or PC-2, due to mutations in their respective *PKD* genes, lead to the deregulation of calcium homeostasis, mediated by the cAMP pathway. In one scenario, a complex of both polycystins could act as a mechanoreceptor signaling via Ca^2+^ influx through PC-2, a TRP channel, after fluid flow registration by PC-1 [7]. On the basis of recent results, the polycystin complex is associated with various cell signaling pathways (c-Myc, Wnt/β-catenin, hedgehog) [8–10]. Looking for new prognostic biomarkers as well as therapeutic targets and procedures is the constant ongoing approach [11–15].

Disease severity in ADPKD is directly related to the gene mutated and to the type of mutation found. Truncating variants of the *PKD1* gene lead to ESRD at approximately 55.6 years of age; whereas nontruncating *PKD1* mutations at 67.9 years of age. In the case of *PKD2* variants, the average age of ESRD is approximately 79.7 years [16, 17]. However, the phenotypic spectrum is often significantly variable within a single family, and ranges from rare severe in utero-onset cases to patients with a preserved glomerular filtration rate into old age. This variability cannot be solely explained by the genetic heterogeneity caused by the *PKD1* and *PKD2* genotypes. Pronounced genic effects upon the clinical manifestation of ADPKD (early onset) have been observed in cases of contiguous deletion of *PKD1* and *TSC2* (OMIM *191092), in cases of mosaicism, and/or in cases of bilineal inheritance of a pathogenic *PKD1* and *PKD2* variant [18–20]. The latest clinical observations and animal studies have stressed the importance of hypomorphic alleles, which individually or in combination with other genes, can influence the severity of the ADPKD phenotype [21].

On the basis of current investigations, very mild PKD phenotypes, particularly in connection with significant polycystic liver disease (PLD), might be the result of a mutated *GANAB* gene (OMIM *104160; coding for glucosidase II subunit α), whose involvement is estimated at approximately 0.3% of total ADPKD [22].

Herein, we report a case of an ADPKD family, in which the youngest male member was referred to the department of nephrology in the age of twenty with an uremic syndrome and ESRD due to ADPKD, and in which bilineal inheritance of pathogenic *PKD1* and *PKD2* variants has been revealed based on genetic studies. We characterize the genic background in the proband and his family members, and discuss the genotype-phenotype correlations.

## Case presentation

**Fig. 1:**
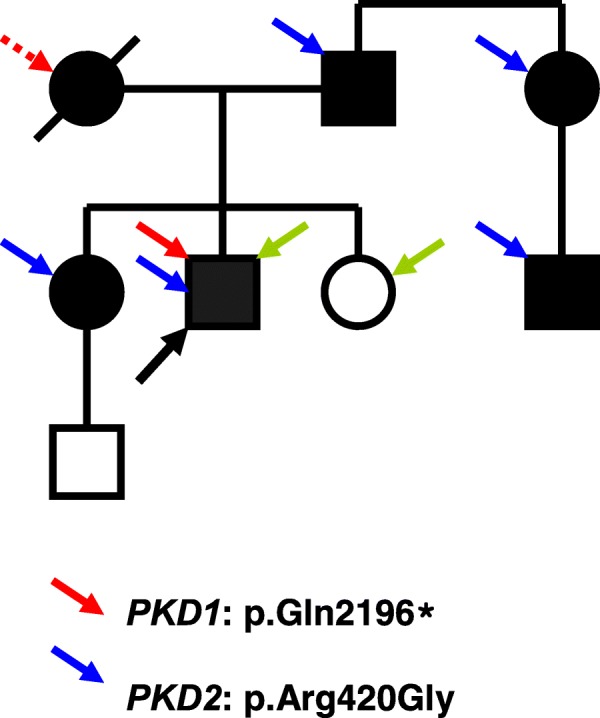
Segregation of the disease in a family. The black-filled individuals, suffering from ADPKD, are marked with a blue arrow (the carriers of *PKD2* mutation), and a red arrow (the carrier of *PKD1* mutation). The dead woman with a dashed red arrow is the expected carrier of *PKD1* mutation. The green arrow indicates the two members of the family with moderately lowered mental abilities. The proband is marked with a black arrow

The proband presented soon after birth with enlarged kidneys filled with multiple cysts. A diagnosis of ADPKD was established based on the clinical picture and positive family history (ADPKD had been found in both father and older sister) (Fig. 1). At the age of 2 he experienced renal colic and acute renal failure, successfully treated conservatively. From the age of 6 he suffered from arterial hypertension, and at the age of 14 chronic renal insufficiency developed. Chronic kidney disease progressed to chronic renal failure at the age of 23. At that age he started hemodialysis treatment and was put on the waiting list for transplantation. After 1 year of hemodialysis treatment he underwent a successful cadaveric kidney transplantation with the graft’s function lasting over 15 years. After this period, he has returned to chronic dialysis treatment. Except for the renal symptoms related to ADPKD, the proband presented with local gigantism (macrodactyly) of the second toe on the left lower limb, which was significantly enlarged. Concurrently, the mental abilities of the proband and his younger sister (without ADPKD) are moderately below-average (they both attended a special school in childhood). Nevertheless, they have achieved writing and reading skills, and displayed sufficient mental and communicative capacities to integrate relatively smoothly into their milieu. Lowered intelligence was not observed in the case of the proband’s older sister, suffering from a mild form of ADPKD. The proband’s 80 year-old father suffers from moderate renal insufficiency (chronic kidney disease stage 3b, eGFR 33 ml/min/m^2^ according to CKD EPI formula) because of polycystic kidneys. He has cysts in his liver, as well. On echocardiography, significant pulmonary and tricuspidal insufficiency was described, with intermittent symptoms of right cardiac failure. The proband’s 52 year-old sister is being treated for polycystic kidneys and liver. Although she was repeatedly hospitalized for infections of renal cysts, her renal function is normal (eGFR 93.6 ml/min/m^2^).

The genomic DNA of all available family members was isolated from peripheral blood lymphocytes following standard procedures. At first, the mutational screening of the *PKD2* gene was carried out within the running scientific project. Because of the mild clinical course of all affected living members (except for the proband), the presence of a *PKD2* causative mutation in the family was highly expected. Mutation analysis was performed as previously described, using heteroduplex analysis and Sanger sequencing [23]. Indeed, a novel missense *PKD2* mutation p.Arg420Gly was found in the proband, who was already dialyzed at that time (reference sequence NM_000297.3). The same mutation was confirmed in other available affected family members (the proband’s father, proband’s aunt, and her son), and later also in the proband’s older sister (see Fig. 1). The co-segregation of mutation with the disease in the family was proven and concurrently tested in 100 unrelated individuals, where no positive capture was observed. The substitution was tested using the verifying methods available to date, and defined as pathogenic.

Unfortunately, the finding of the *PKD2* mutation couldn’t explain the serious ADPKD phenotype of the proband. For that reason, the seeking out of other affected family members was initiated. Finally, it was discovered that the proband’s mother had already died of renal failure at the age of 45 because of unrecognized polycystic kidney disease only established post mortem. Unfortunately, the DNA of the proband’s dead mother was not available for further examination; this also was the case for the DNA of the other living affected family members, because the proband’s mother had come from an orphanage. Nevertheless, concerning the early death of the proband’s mother, it was highly probable that she suffered from ADPKD type 1, and thus passed the pathogenic *PKD1* sequence variant on to her son. Therefore, a mutational analysis of *PKD1* of the proband was performed in order to identify the causative mutation of this gene. The experimental strategy was based on long-range PCR, followed by nested PCR and High Resolution Melting analysis (HRM). All nested PCR fragments showing aberrant melting curves on HRM were verified by Sanger sequencing in both directions. The entire procedure (including primers and PCR reaction conditions) was described in detail by Reiterová et al. [19]. As a result, the novel nonsense *PKD1* mutation p.Gln2196* with a definitely pathogenic effect was identified (reference sequence NM_001009944.2). This mutation was subsequently published by Obeidova et al. [24]. All available family members (together with the proband’s older sister) were tested for the presence of this new *PKD1* mutation; however, it was not found in any of them. The proband was thus the only living person in this family who carried the pathogenic nonsense *PKD1* mutation. Besides, he also possessed the previously identified causative *PKD2* mutation. Co-inheritance of both of these sequence variants of both *PKD* genes, and their common impact on ADPKD manifestations, are the presumptive explanation for such a complicated clinical course.

Interestingly, the same nonsense *PKD1* mutation (p.Gln2196*) recently has been identified in another Czech family (in three women from three generations). The age of ESRD in the first and the second generation were 63 and 51, respectively. The third generation patient (32 years old) is being treated for hypertension. No additional PKD sequence variant has been found in this family up to the present. Some relationship between both of the families mentioned can not be ruled out.

The lower mental capabilities of the proband and his younger sister suggest a possible genetic load within the family. To solve this hypothesis, a Multiplex Ligation-dependent Probe Amplification (MLPA) analysis was carried out using a SALSA MLPA P245 Microdeletion Syndrome-1 kit, a SALSA MLPA P036 Subtelomeres Mix 1 kit, and a SALSA MLPA P070 Subtelomeres Mix 2B kit (all from MRC-Holland). The results of the MLPA were carried out by fragmentation analysis with the use of an ABI PRISM® 3130 Genetic Analyzer (Applied Biosystems). To obtain the final results, the raw data generated were normalized according to the manufacturer’s instructions. An in-house developed method was used for all calculations. The MLPA analysis was done with the DNA of our proband, and with the DNA of his older sister with normal intelligence (negative control). The DNA of the proband’s younger sister, with lower mental abilities (but without ADPKD) was unfortunately unavailable. The results of all three MLPA analyses were negative in both samples; no deletion or any other aberration was detected. As another step in our examination, the DNA of the proband was put through whole-genome screening by the method of Comparative Genomic Hybridization (array CGH) (Agilent, SurePrint G3 CGH ISCA v2 Microarray 8x60K) with the following specifications: CytoGenomics Edition 4.0.3.12, Agilent software; whole-genome screening with 240 kb resolution (48 kb in ISCA); Human genome GRCh37/hg19 database. As in the case of MLPA, no significant deletion/duplication referring to the corresponding phenotype was discovered. However, several large-scale studies have demonstrated that array CGH has a 10–20% detection rate of chromosomal abnormalities in children with mental retardation/developmental delay, with or without congenital anomalies [25]. Thus, a hereditary origin of the low mental abilities of the proband and his younger sister still can’t be excluded.

## Discussion and conclusions

For the first time, within the Czech cohort of patients, we report on the bilineal inheritance of a *PKD1* and *PKD2* mutation within a family with ADPKD. The *PKD1* and *PKD2* genes, which are two basic genetic determinants of ADPKD, were localized in the early ‘90s of the previous century [4, 5]. Recently, the real genetic background of this disease has been found to be much more complicated. ADPKD shows significant inter- and intrafamilial phenotypic variability in the rate of disease progression and extra-renal manifestations, which can hardly be explained by the presence of single pathogenic mutation in the causal gene. In the vast majority of cases, mild phenotypes are related to the *PKD2* gene. On the other hand, severe ADPKD manifestations, already frequently diagnosed in utero or at birth, have also been described [21, 26, 27]. These findings not only suggest the role of the *PKD1* and *PKD2* causative genes themselves, but also the involvement of heritable so-called modifier genes. Thus, the most severe PKD phenotypes are supposed to be a consequence of the concurrent impact of two co-inherited mutations; the first in the causative *PKD1* gene, and the second in a modifier gene [20, 28]. Moreover, the second mutation of the modifier gene itself need not necessarily have the causative effect; a similar impact on the resulting deteriorated condition of an actual patient (family) may have the presence of either a hypomorphic allele (incompletely penetrant allele or allele, causing only partial loss of gene function) or an allele carrying polymorphisms or sequence variants of so far unknown significance (and acting both in cys or in trans) [19, 21, 26, 29, 30]. The common effect of two *PKD1* hypomorphic alleles (in a homozygous or in a compound heterozygous state) on the development of ADPKD has also been described [31]. All of the main causative genes concerning polycystic kidney disease in general (*PKD1*, *PKD2*, *PKHD1* as the main determinant of ARPKD or *HNF-1β*) in some circumstances can act as modifier genes and/or hypomorphic alleles [32]. Different combinations of causative and modifier genes thus probably ensure the enormous variability of ADPKD phenotypes. In reference to the *PKD1* gene in general, the inherited modifiers and genetic background are supposed to account for an estimated 18 to 59% of the phenotypic variability [33].

In the case of the patient reported in this present publication, both mutations found in the *PKD1* and *PKD2* genes were described as highly likely to be pathogenic, and thus both with the highest probability have a causative effect [23, 24]. In the first place, the nonsense *PKD1* mutation p.Gln2196* (probably inherited from the proband’s mother) is responsible for the unfavorable ADPKD type 1 phenotype, which is strongly aggravated by the presence of the second missense *PKD2* mutation p.Arg420Gly. Despite the fact that we unfortunately don’t have a precise clinical characterization of the proband’s mother, as probably the only family representative simply having a *PKD1* mutation alone, the disease manifestation in the proband is demonstrably much worse than the ordinary phenotype of ADPKD type 1 (ESRD at the age of 23 in the case of the proband, versus mid fifties for ADPKD type 1 in general). The finding of the second Czech family with a p.Gln2196* mutation of *PKD1* (ESRD at the age of 63 and 51) is in full accordance with this assumption.

The first documentation of bilineal disease in ADPKD was published by Pei et al. in 2001, and completed by the same author 11 years later [20, 34]. There the authors described two ADPKD patients (members of a multigeneration family) with trans-heterozygous mutations in both the *PKD1* and *PKD2* genes. Molecular analysis of *PKD2* revealed frameshift mutation p.Asn720fs; the presence of the second pathogenic mutation within *PKD1* (p.Tyr528Cys) was proven later on, as itself causing a mild ADPKD phenotype. Also, in the case of these two patients with bilineal inheritance (ESRD at the age of 48 and 52), their ADPKD phenotype was more severe in comparison with other family members carrying only one type of PKD mutation. The clinical testing of two patients with trans-heterozygous *PKD1* and *PKD2* mutations showed severely reduced creatinine clearance when compared with patients who had only the *PKD1* mutation, as well as more liver cysts than other affected family members, who had either *PKD1* or *PKD2* mutations alone. However, none of the two patients discussed had massive polycystic liver, resulting in either portal hypertension or hepatic failure. Also, none of the affected family members had any evidence of intracranial arterial aneurysms.

Another case of co-inheritance of the *PKD1* and *PKD2* mutation in trans was reported by Dedoussis et al. [28]. The proband of this study was a woman, diagnosed at the age of 39, suffering from hypertension and multiple cysts in both kidneys (without extrarenal cysts and without vascular complications and aneurysms). She reached ESRD at the age of 41, and at the age of 51 underwent successful kidney transplantation (from a cadaver donor). Again, ADPKD manifestation of this proband is thus demonstrably more serious than ADPKD caused by only a single mutation of *PKD1*. Detailed genetic analysis of the proband’s DNA revealed a novel homozygous missense mutation of *PKD2* (p.Phe482Cys) and a de novo heterozygous *PKD1* splice-site mutation (c.8017-2_-1delAG in IVS21). The pathogenicity of the novel *PKD2* substitution was backed up with some experimental studies concerning protein (PC-2) expression, localization, and function.

The disease-modifying role of the non-causative missense variant was recently demonstrated by Ali et al. [30]. In that report, it was the *PKD1* itself, which played the role of causative gene, and at the same time the role of modifying allele with a non-causative sequence variant acting in trans. In the four-generation’s family, the nonsense *PKD1* mutation p.Gln2243* was determined, which co-segregated with the disease in the family, showing the typical ADPKD type 1 phenotype. Two members of that family (brothers), except for the nonsense mutation mentioned above, carried another *PKD1* sequence variant (p.His1769Tyr), evaluated as a variant of undetermined significance using different computer prediction tools. Both brothers inherited this new sequence variant from their healthy father. The first proband carrying both the causative and the undetermined *PKD1* sequence variant had already reached ESRD at the age of 29. His brother, with the same *PKD1* genotype, showed a similar extreme enlargement of the kidneys, although his renal function was in the normal range. The undetermined sequence variant itself had only a very mild effect on disease manifestation, as was indicated by the case of the healthy father of the two affected brothers. The father carried only the undetermined variant without any *PKD1* causative mutation. Although he was considered to be healthy, had normal kidney volume, normal kidney function, and no family history of ADPKD, an ultrasound examination revealed seven renal cysts at the age of 58.

A similar case of a bilineal ADPKD family with two *PKD1* mutations/sequence variants in trans (in-frame deletion and substitution) has been recently reported by Hwang et al. [35].

Not only the genes directly determining the polycystic kidney disease (ADPKD or ARPKD) can act as modifiers in the development of ADPKD. The modifying effect and related aggravated disease phenotype also has recently been described in connection with the *TTC21B* gene determining other kinds of ciliopathies (viz.*,* nephronophthisis and focal segmental glomerulosclerosis) [36].

More genetic variants elsewhere in the genome, modifying the progression of ADPKD, could be further revealed by current methods of molecular genetics, such as Next-Generation Sequencing (NGS) or Genome-Wide Association Study (GWAS) [29, 37–39].

In conclusion, here we described the first ADPKD family in the Czech Republic, with an atypical clinical course of one family member, caused by bilineal inheritance of pathogenic *PKD1* and *PKD2* variants. The presented report illustrates the fact that not only the single gene (*PKD1* or *PKD2*) is responsible for the manifestation of ADPKD. Except for the causative gene, the final ADPKD phenotype is also modulated by renal or alternatively non-renal modifier genes, hypomorphic alleles, alleles carrying different (especially SNP) polymorphisms, and by the genetic background, in general. Co-inheritance of two or more genes/alleles in connection with ADPKD appears not to be as rare as previously considered. On the contrary, this is probably one of the main mechanisms explaining the existing vast inter- and intrafamilial variability in the ADPKD phenotype.

## Abbreviations

ADPKD: Autosomal dominant polycystic kidney disease
ARPKD: Autosomal recessive polycystic kidney disease
CGH: Comparative genomic hybridization
ESRD: End stage renal disease
HRM: High resolution melting
MLPA: Multiplex ligation-dependent probe amplification
PC-1: Polycystin 1
PC-2: Polycystin 2
PKD: Polycystic kidney disease
PLD: Polycystic liver disease
